# Development and application of a multiplex TaqMan real-time PCR assay for detecting pathogenic fungi causing root rot in red kidney bean

**DOI:** 10.3389/fpls.2025.1720901

**Published:** 2025-12-19

**Authors:** Wenbo Liu, Xurong Peng, Hanwei Li, Huiting Kang, Jie Zhao, Chun Yang, Xiaojun Zhao, Xiaojuan Hao

**Affiliations:** 1 Shanxi Key Laboratory of Bioagent Utilization and Eco-Pesticide Innovation, College of Plant Protection, Shanxi Agricultural University, Jinzhong, China; 2 Shanxi Key Laboratory of Integrated Pest Management in Agriculture, College of Plant Protection, Shanxi Agricultural University, Taiyuan, China

**Keywords:** *Fusarium oxysporum*, *Fusarium solani*, *Fusarium tricinctum*, multiplex TaqMan real-time PCR, red kidney bean

## Abstract

*Fusarium* root rot poses a significant threat to red kidney bean (*Phaseolus vulgaris*) production in Shanxi Province, China, and is primarily caused by *Fusarium oxysporum*, *Fusarium tricinctum*, and *Fusarium solani*. Currently, no method exists for the rapid and simultaneous detection of these three pathogens. In this study, we developed a multiplex TaqMan real-time PCR assay targeting the translation elongation factor 1-alpha (TEF-1α) gene. Specific primers and probes were designed based on conserved regions within TEF-1α. The assay demonstrated high specificity and sensitivity, with detection limits of 1.46 × 10³, 7.17 × 10³, and 1.76 × 10² copies·μL^-1^ for *F. oxysporum*, *F. tricinctum*, and *F. solani*, respectively. Intra- and inter-assay variability tests showed high reproducibility, with coefficients of variation below 2%. Field surveys conducted in Shanxi’s main production areas assessed the root rot disease index, and corresponding soil samples were collected. A logistic regression model was established to predict disease index based on the total DNA copy number of the three pathogens per gram of soil: y= 100−96.670/ [1 + (x/7.253)^9.350^] (R^2^ = 0.927). This multiplex TaqMan real-time PCR assay provides a rapid, specific, and reproducible tool for simultaneous detection of these *Fusarium* pathogens, supporting early disease intervention and reducing crop losses in red kidney bean.

## 1 Introduction

Red kidney bean (*Phaseolus vulgaris*), a legume valued for its high nutritional content, exhibits strong drought tolerance and a short growth cycle, making it an economically important crop worldwide (Koide et al., 1997; Li et al., 2024). Since its introduction to Shanxi Province, China, from Italy in the 1990s, it has become a major cash crop, leading the nation in both cultivation area and export volume since 2017 (Hu et al., 2017; Guo et al., 2024). However, over the past decade, the intensification of continuous monocropping has led to an increasing incidence of root rot, a destructive soil-borne fungal disease that severely impacts production (Zhang et al., 2021; Li et al., 2025). Infection by *Fusarium* species typically causes root and crown rot, leading to taproot decay, stunted growth, leaf yellowing, and wilting. Annual yield losses in Shanxi range from 30% to 80%, posing a substantial threat to the local kidney bean industry (Guo et al., 2024). While *F. solani* was previously identified as the main pathogen in the region (Liu, 2012), our group has further demonstrated that *F. oxysporum* and *F. tricinctum* also act as predominant pathogens, frequently occurring as complex infections (Zhai, 2020).

Early and accurate detection of soil-borne pathogens is essential for implementing timely control strategies and mitigating crop losses (Wang and Liu, 2024). Conventional fungal identification methods often entail laborious procedures, prolonged incubation periods, and potential taxonomic inaccuracies (Arbefeville et al., 2017). Molecular diagnostic approaches overcome these constraints by enabling precise pathogen detection at the nucleic acid level, often prior to symptom development (Arbefeville et al., 2017). Such early and reliable identification is crucial for guiding targeted disease management and curbing pathogen dissemination (Prusky, 1996; Dean et al., 2012).

Polymerase Chain Reaction (PCR) represents the most widely used molecular technique for microbial species identification (Ozbek et al., 2009). However, conventional PCR does not provide immediate analytical results, as it requires additional post-amplification procedures, including electrophoretic separation and amplicon sequencing, to facilitate data interpretation (Malarczyk et al., 2020). To address these limitations, several advanced PCR-based methods have been developed. Among them, real-time quantitative PCR (qPCR) allows for simultaneous amplification and quantification of target genes, eliminating the need for post-reaction processing and substantially reducing turnaround time (Chen et al., 2010). Furthermore, the TaqMan probe system can be readily adapted to multiplex qPCR assays, enabling simultaneous quantification of several pathogens in a single reaction, which significantly enhances detection throughput and operational efficiency (Livak et al., 1995; Reich et al., 2016). These qPCR-based approaches have been extensively applied in the detection of soil-borne fungal pathogens, supporting disease diagnosis, epidemiological studies, and timely management interventions (Atallah et al., 2007; Kim and Knudsen, 2008; Ziesman et al., 2016; Milner et al., 2019). Isothermal amplification methods, such as loop-mediated isothermal amplification (LAMP) (Kong et al., 2020), recombinase polymerase amplification (RPA) (Xi et al., 2024), and rolling circle amplification (RCA) (Banér et al., 1998), have also gained attention due to their minimal instrumentation requirements and operational simplicity. Nevertheless, their dependence on specialized enzymes and complex primer design continues to pose challenges for multiplexing and large-scale application. Despite these challenges, the continuous evolution and complementary application of molecular diagnostics, from qPCR to isothermal methods, are fundamentally driving progress in the field. Consequently, plant disease management has advanced beyond simple pathogen identification toward integrated decision-support systems that merge quantitative detection, epidemiological modeling, and field management. The development of “detection-prediction” platforms—by integrating sensitive molecular assays with predictive models—has therefore emerged as a critical pathway to enable precise early warning and sustainable control strategies.

The translation elongation factor 1-alpha (TEF-1α) gene robustly discriminates *Fusarium* species, including cryptic lineages within species complexes (Ralf et al., 2005). Its utility stems from conserved exon-flanking primers that amplify hypervariable introns, which exhibit exceptional interspecific polymorphism (Rehner and Buckley, 2005). Consequently, these TEF-1α-based markers are widely implemented in qPCR assays for rapid and specific detection (Sohlberg et al., 2022).

In this study, a set of specific probes based on TEF-1α was designed for the detection of three fungal pathogens (*F. oxysporum*, *F. tricinctum* and *F. solani*) infecting red kidney bean plants. Subsequently, an efficient multiplex TaqMan real-time PCR assay was developed to simultaneously detect and differentiate these fungi species. The relationship between the levels of the three pathogenic *Fusarium* species in the soil and the incidence of red kidney bean root rot was determined. This provides a theoretical basis for diagnosing, monitoring, and preventing red kidney bean root rot.

## 2 Materials and methods

### 2.1 Materials

The pathogenic fungal strains employed in this study were characterized and maintained by our research group (**Table 1**). Field surveys for disease incidence were conducted in 45 fields across major red kidney bean production areas of Shanxi Province. Soil samples were collected from each field site, placed in sterile plastic bags, and stored at −20°C (**Table 2**).

**Table 1:** Fungal isolates employed in this study.

Pathogen Name	Isolate code	Hosts
*Fusarium oxysporum*	K5-21	*Phaseolus vulgaris*
*Fusarium tricinctum*	K3-32	*Phaseolus vulgaris*
*Fusarium solani*	R2-4-1	*Phaseolus vulgaris*
*Fusarium equiseti*	K7-47	*Phaseolus vulgaris*
*Fusarium proliferatum*	K3-113	*Phaseolus vulgaris*
*Fusarium redolens*	K1-1	*Phaseolus vulgaris*
*Fusarium verticillioides*	S1-11-1	*Phaseolus vulgaris*
*Fusarium chlamydosporum*	K4-88	*Phaseolus vulgaris*
*Fusarium graminearum*	FG-Y	*Zea mays*
*Setosphaeria turcica*	ST-Y	*Zea mays*
*Fusarium graminearum*	FG-M	*Triticum aestivum*
*Alternaria solani*	AS-F	*Solanum lycopersicum*
*Botrytis cinerea*	BC-C	*Fragaria ananassa*
*Phytophthora*	PS-Y	*Prunus persica* var. *nectarina*
*Colletotrichum gloeosporioides*	CG-P	*Malus pumila*

**Table 2:** Collection information for soil samples in this study.

Code number	Hosts	Locations
1-3	Red kidney bean	Yangqu County, Taiyuan, Shanxi, China
4-9	Red kidney bean	Jingle County, Xinzhou, Shanxi, China
10	Red kidney bean	Wuzhai County, Xinzhou, Shanxi, China
11-22	Red kidney bean	Shenchi County, Xinzhou, Shanxi, China
23-35	Red kidney bean	Kelan County, Xinzhou, Shanxi, China
36-45	Red kidney bean	Lan County, Lvliang, Shanxi, China

### 2.2 DNA extraction

After 7d of incubation in the dark on potato dextrose agar (PDA) at 25°C (thermostatic incubator, Shanghai Boxun Equipment Factory, China), about 50 mg of mycelium was gently scraped off the surface of the agar. Genomic DNA was then extracted from the harvested mycelium using the CTAB method (Ristaino et al., 1998). For soil samples, 0.25 g aliquots (**Table 2**) underwent pretreatment with Humic Acid-Be-Gone A Solution (Sangon Biotech, Shanghai, China) to facilitate humic acid removal, prior to DNA extraction with a Rapid Soil DNA Isolation Kit (Sangon Biotech). Genomic DNA from plant tissues was obtained using the Rapid Plant Genomic DNA Isolation Kit (Sangon Biotech), in accordance with the manufacturer’s instructions.

### 2.3 Design and synthesis of TaqMan probes

The TEF-1α-based species-specific primers previously developed by our group for *F. oxysporum*, *F. tricinctum*, and *F. solani* were employed without modification (Xue, 2022; Ma, 2024). Based on these specific primers, TaqMan probes for the three *Fusarium* species were subsequently designed using Beacon Designer 8 software (**Table 3**). All primers and probes were synthesized by Sangon Biotech and dissolved in ddH_2_O.

**Table 3:** Primers and probes of *F. oxysporum*, *F. tricinctum*, and *F. solani*.

Pathogen	Primer and probe	Sequence (5’-3’)	Length (bp)
*F. oxysporum*	JB-2F	CGTTTGCCCTCTTACCAT	86
	JB-2R	CAGCGGCTTCCTATTGTT	
	JB-2Probe	HEX-CCTCAATGAGTGCGTCGTCACGTG-BHQ1	
*F. tricinctum*	Fus-F	GGTCACTTGATCTACCAGT	135
	Ftr-R	TGGATGCGTTTCGAGTGA	
	Ftr-Probe	FAM-TTACGCGCGCTCCCATCGATTCC-BHQ1	
*F. solani*	FP-4F	CGCTAACCGGTCCAACAATAG	196
	FP-4R	GGTGAGACATGTGTGAGAGAGGT	
	FP-4Probe	CY5-CCGCTGAGCTCGGTAAGGGTTCCT-BHQ2	

### 2.4 Optimization of the reaction conditions for multiplex TaqMan real-time PCR

The sequences of primers and probes utilized in this study are presented in **Table 3**. All qPCR assays were conducted using a Bio-Rad CFX96™ Real-Time System (Bio-Rad, USA). The uniplex real-time PCR assay targeting *F. oxysporum*, *F. tricinctum*, and *F. solani* was conducted in a total reaction volume of 20 µL. The reaction mixture comprised 10 µL of TaqProbe 2× qPCR-Multiplex Mastermix (Sangon Biotech), 0.4 µL each of forward and reverse primers (10 µmol·L^-1^), 0.4 µL of TaqMan probe (10 µmol·L^-1^), 2 µL of DNA template, and sterile double-distilled water to reach the final volume. The thermal cycling conditions were as follows: initial denaturation at 95°C for 30 s; followed by 40 cycles of denaturation at 95°C for 15 s and annealing/extension at 60°C for 30 s. The multiplex real-time PCR assay was developed from established uniplex assays by systematic optimization of critical reaction parameters. Annealing temperature and primer/probe concentrations were specifically optimized using a temperature gradient (52.0, 52.7, 54.0, 55.9, 58.4, 60.3, 61.4, and 62.0°C) and a concentration series (0.2, 0.4, 0.6, 0.8, and 1.0μmol·L^-1^), respectively. The optimal PCR conditions were determined by evaluating amplification efficiency, as reflected in the quantification cycle (Cq) values and fluorescence intensity measurements. All reactions were conducted in triplicate to ensure reproducibility.

### 2.5 Construction of standard plasmids and standard curves

The standard DNA fragments of target fungi were amplified through conventional PCR assays using primers listed in **Table 3** (JB-2F/JB-2R for *F. oxysporum*, Fus-F/Ftr-R for *F. tricinctum*, FP-4F/FP-4R for *F. solani*). The PCR amplification was performed in a 25 μL reaction mixture containing 12.5 μL of 2× Taq Master Mix (Coolaber Technology Co., Ltd., Beijing, China), 1 μL of each primer (10 µmol·L^-1^), 1 μL of DNA template, and 9.5 μL of sterile ddH_2_O. The thermal cycling procedure comprised an initial denaturation step at 94°C for 4 min, followed by 35 amplification cycles consisting of denaturation at 94°C for 30 s, annealing at 60°C for 30 s, and extension at 72°C for 1 min. The protocol concluded with a final extension phase at 72°C for 10 min. The PCR amplification products were resolved via electrophoresis on a 2.5% agarose gel prepared with 1× TAE buffer. Subsequently, these products were ligated into the pMD18-T vector (TaKaRa, Beijing, China) to construct recombinant plasmids, which were then subjected to sequencing analysis by Sangon Biotech Co., Ltd. (Shanghai, China). Plasmid DNA concentrations were quantified using BioDrop μLite+ ultra-micro spectrophotometers (Biochrom Ltd., Cambridge, United Kingdom), and the copy number was calculated according to Lamien et al. (2011) as follows:


$$number\;of\;copies=\frac{\;\left[amount\;\left(ng\right)\;\times \;6.022\;\times \;10^{23}\right]}{\left[length\;\left(plasmid\;+\;insert\right)\;\times \;1\;\times \;10^{9}\;\times \;650\right]}$$

Standard curves were generated for each target using serial ten-fold dilutions of the corresponding recombinant plasmids.

### 2.6 Specificity of the multiplex TaqMan real-time PCR assay

To assess the specificity of the multiplex TaqMan real-time PCR assay, genomic DNA from representative strains (**Table 1**) was amplified using this system. DNA from *F. oxysporum*, *F. tricinctum*, and *F. solani* served as positive controls, with sterilized ddH_2_O employed as a negative template control.

### 2.7 Sensitivity of the multiplex TaqMan real-time PCR assay

To determine the analytical sensitivity of the multiplex TaqMan real-time PCR assay, ten-fold serial dilutions of recombinant plasmids for *F. oxysporum* (1.46 × 10^9^ to 1.46 × 10^0^ copies·μL^-1^), *F. tricinctum* (7.17 × 10^9^ to 7.17 × 10^0^ copies·μL^-1^), and *F. solani* (1.76 × 10^9^ to 1.76 × 10^0^ copies·μL^-1^) were utilized as templates. Reactions yielding Cq values greater than 35 were defined as negative.

### 2.8 Repeatability analysis

The reproducibility of the multiplex TaqMan real-time PCR assay was evaluated using standard plasmids at three distinct concentrations (10^7^, 10^5^, and 10³ copies·μL^-1^). Both intra-assay and inter-assay variability were assessed by performing triplicate measurements of each concentration on separate days. The results were quantified using the coefficient of variation (CV).

### 2.9 Establishment of a logistic regression model relating soil *Fusarium* abundance to root rot disease index in red kidney bean

The multiplex TaqMan real-time PCR assay was applied to quantify *Fusarium* abundance in forty-five field rhizosphere soil samples (**Table 2**), with each sample analyzed in triplicate. Mean Cq values from technical replicates were used to estimate the soil abundance (per gram of soil) of *F. oxysporum*, *F. tricinctum*, and *F. solani*. The total *Fusarium* load per sample was calculated as the sum of these three species. A four-parameter logistic (4PL) regression model was then fitted to characterize the relationship between the log-transformed total *Fusarium* DNA copy number (independent variable, *x*) and the corresponding root rot disease index (dependent variable, *y*). The 4PL model is well-established in plant disease epidemiology for describing sigmoidal disease progress curves (Campbell and Madden, 1990; Hau, 1993), and its flexibility allows parameters to be constrained based on biologically realistic bounds (Paul and John, 2005). In this study, the upper asymptote (*A_1_*) was fixed at 100, reflecting the theoretical maximum disease index, while the lower asymptote (*A_2_*) was set to 3.33, corresponding to the experimental detection threshold for pathogen presence—a biologically meaningful alternative to zero. The model was formulated as:


$$y=A_{1}+\frac{A_{2}-A_{1}}{\left[1+{\left(\frac{x}{x_{0}}\right)}^{p}\right]}$$

Where *x_0_* represents the midpoint of the curve and *p* represents the steepness of the curve. The disease index (DI) was calculated according to Wang et al. (2023a) as follows:


$$DI=\frac{\left[\sum \left(number\;of\;plants\;at\;each\;disease\;level\;\times \;relative\;grade\;value\right)\right]}{\left(total\;number\;of\;plants\;investigated\;\times \;maximum\;disease\;grade\;value\right)\;}\times 100$$

Disease index classification criteria are provided in **Table 4**.

**Table 4:** Classification criteria for disease index.

Grade	Description of symptoms
0	There are no disease spots on the stem base or main root.
1	There are a few spots at the stem base and main root.
3	Disease spot area accounts for 1/4~1/2 of total root area.
5	Disease spot area accounts for 1/2~3/4 of total root area.
7	The disease spots form a phenomenon of wrapping around the stem, and the root did not die.
9	The roots are necrotic, and the above-ground parts are withered.

## 3 Results

### 3.1 Optimization of the reaction conditions for multiplex TaqMan real-time PCR

**Figure 1:**
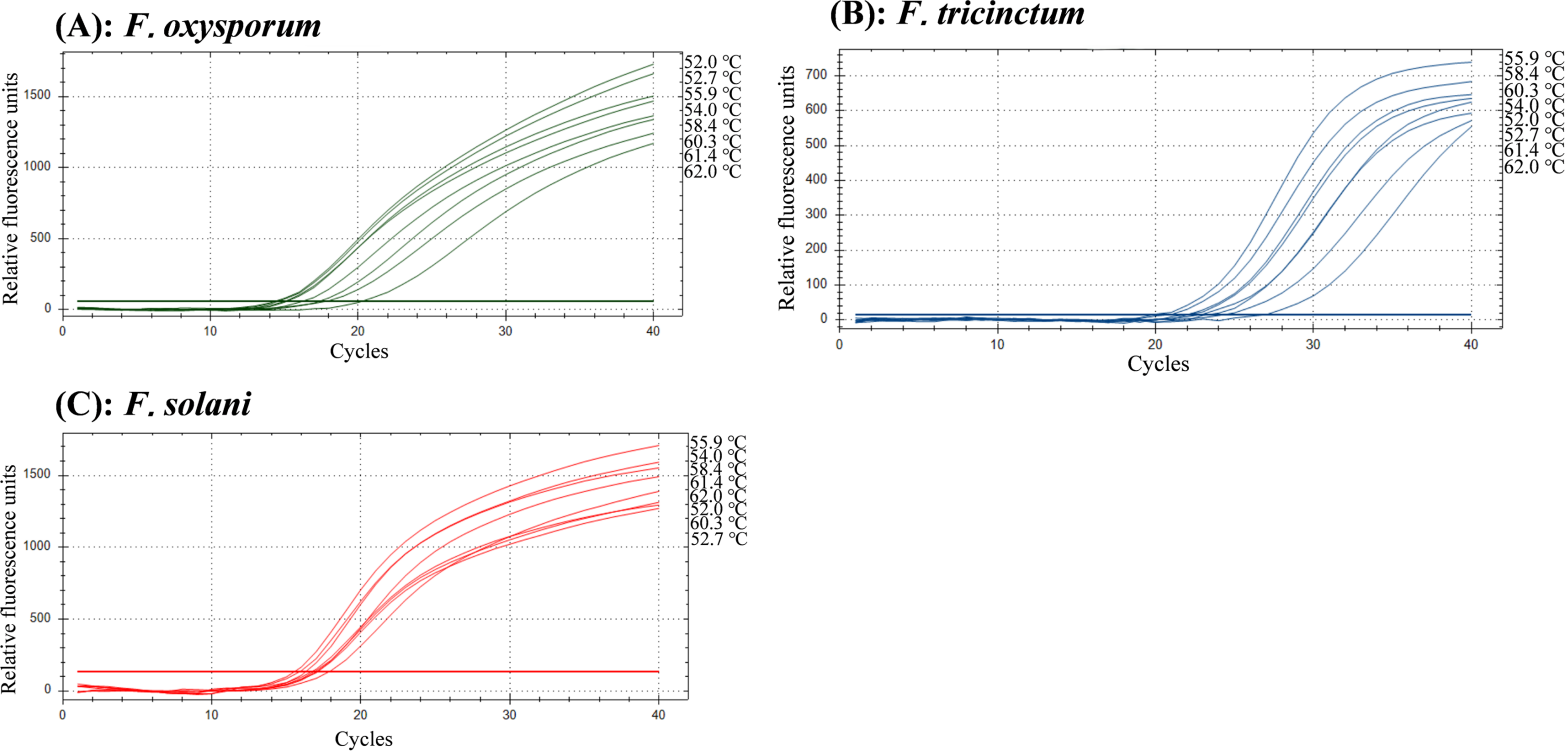
Annealing temperature optimization for the multiplex TaqMan real-time PCR assay detecting *F oxysporum***(A)**, *F tricinctum***(B)**, and *F solani***(C)**.

The optimal primer and probe concentrations for the multiplex TaqMan real-time PCR assay are presented in **Tables 5**–**7**. For *F. oxysporum* and *F. solani*, both the optimal primer and probe concentrations were optimized at 1 µmol·L^-1^. Regarding *F. tricinctum*, the primer concentration was optimized at 1 µmol·L^-1^, while the probe concentration was set at 0.8 µmol·L^-1^. The optimal annealing temperatures for *F. oxysporum*, *F. tricinctum* and *F. solani* were 52.0, 55.9 and 55.9°C, respectively (**Figure 1**). Based on a comprehensive assessment of reaction performance, 55.9°C was determined to be the optimal annealing temperature.

**Table 5:** Optimization of primer and probe concentrations for *F. oxysporum*.

TaqMan probe (µmol·L^-1^)	Forward and reverse primer (μmol·L^–1^)				
	0.2	0.4	0.6	0.8	1.0
0.2	33.61	31.77	25.00	23.46	21.65
0.4	32.75	33.72	29.62	23.47	21.95
0.6	32.08	34.01	30.41	23.04	22.07
0.8	31.18	33.34	32.41	24.05	21.68
1.0	30.59	34.66	27.21	22.21	19.49

The values in the table are Cq averages in cycles;

Concentrations are for primers/probe prior to use.

**Table 6:** Optimization of primer and probe concentrations for *F. tricinctum*.

TaqMan probe (µmol·L^-1^)	Forward and reverse primer (μmol·L^–1^)				
	0.2	0.4	0.6	0.8	1.0
0.2	38.88	38.93	30.32	25.37	23.40
0.4	38.67	38.69	30.25	24.01	20.37
0.6	38.98	38.18	30.90	22.02	20.07
0.8	36.25	37.88	30.33	22.87	19.40
1.0	33.33	28.22	25.67	21.43	19.61

The values in the table are Cq averages in cycles;

Concentrations are for primers/probe prior to use.

**Table 7:** Optimization of primer and probe concentrations for *F. solani*.

TaqMan probe (µmol·L^-1^)	Forward and reverse primer (μmol·L^–1^)				
	0.2	0.4	0.6	0.8	1.0
0.2	23.58	23.49	20.76	18.66	18.53
0.4	21.79	20.08	19.45	18.82	17.01
0.6	21.23	21.13	18.01	18.14	16.05
0.8	20.86	20.25	17.18	18.12	15.70
1.0	20.31	20.17	16.37	17.29	15.02

The values in the table are Cq averages in cycles;

Concentrations are for primers/probe prior to use.

### 3.2 Establishment of standard curves

**Figure 2:**
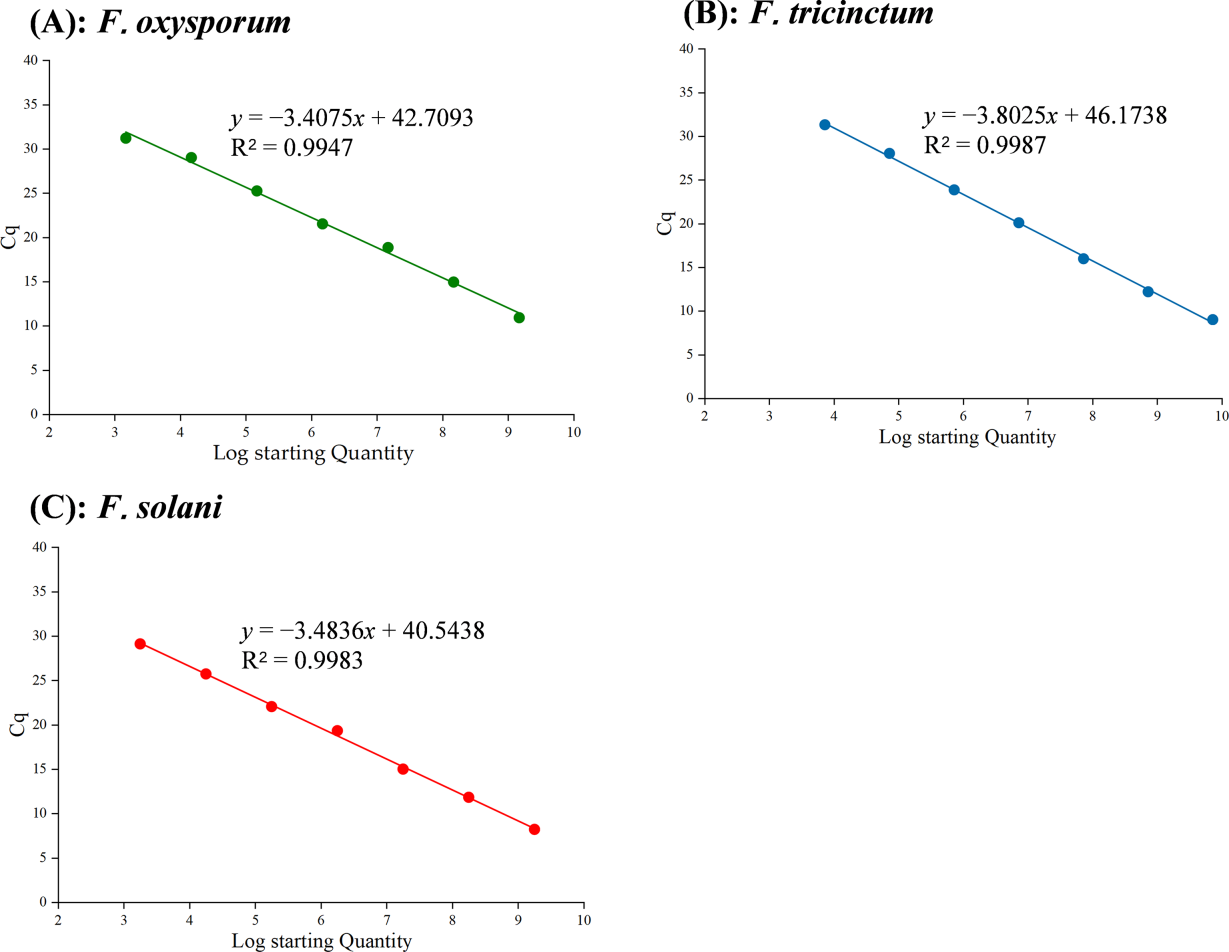
Standard curves obtained by multiplex TaqMan real-time PCR assay for *F oxysporum***(A)**, *F.tricinctum***(B)** and *F solani***(C)**.

The specific primer pairs (**Table 3**) were used to amplify fragments of the expected sizes for *F. oxysporum*, *F. tricinctum*, and *F. solani*. The amplified products were subsequently inserted into the pMD18-T vector (TaKaRa, Beijing, China) to construct recombinant plasmids corresponding to each species. The concentrations of recombinant plasmids for *F. oxysporum*, *F. tricinctum* and *F. solani* were 1.46 × 10^10^, 7.17 × 10^9^ and 1.76 × 10^10^ copies·µL^−1^, respectively. Standard curves were established by performing tenfold serial dilutions of recombinant plasmid samples. All three standard curves exhibited coefficients of determination (R²) greater than 0.99 (**Figure 2**), demonstrating a strong linear correlation between the logarithmic plasmid copy number and the Cq value for each standard.

### 3.3 Specificity of the multiplex TaqMan real-time PCR assay

**Figure 3:**
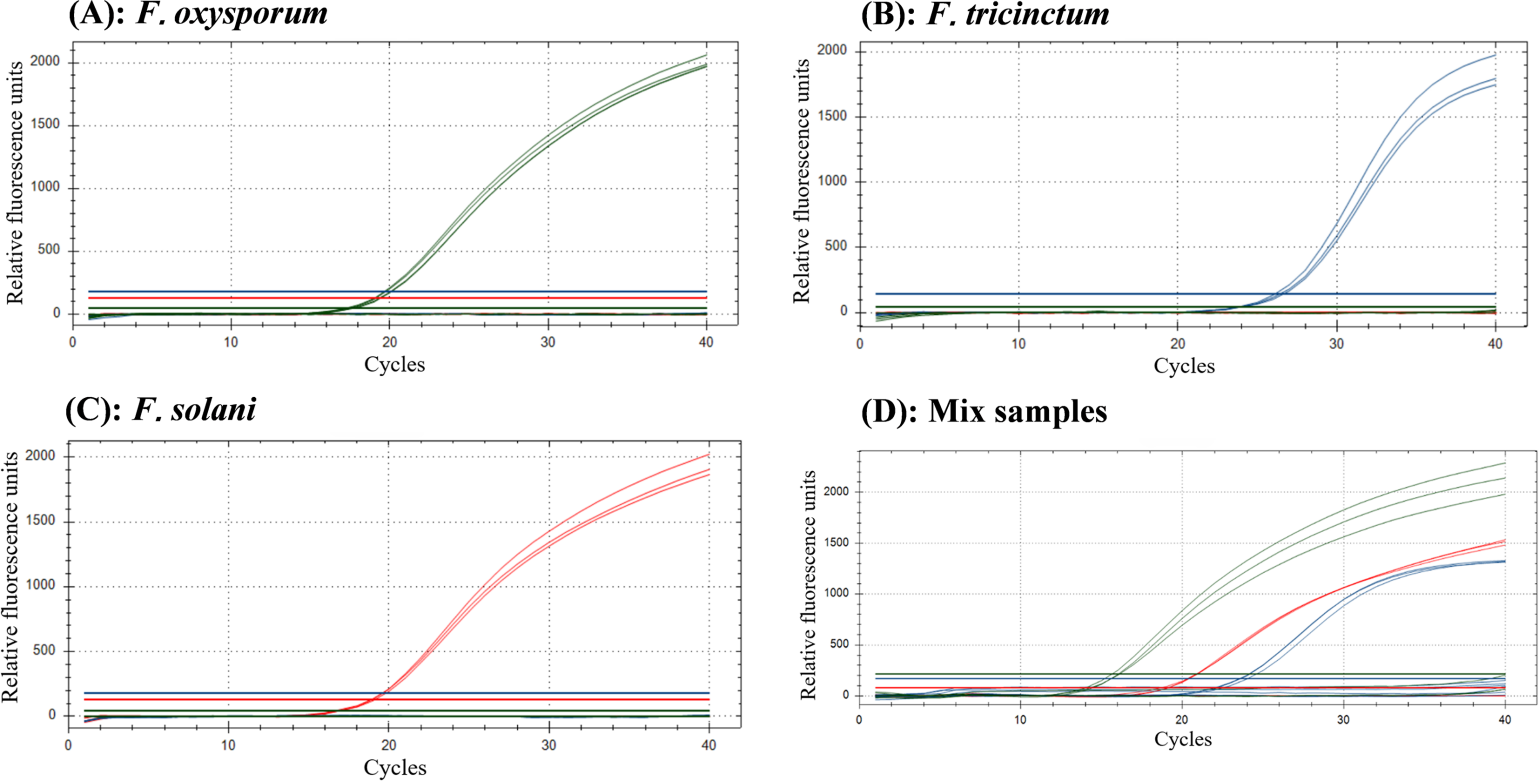
Specificity results of multiplex TaqMan real-time PCR. The specific fluorescent signals were observed in the positive samples of *F oxysporum***(A)**, *F tricinctum***(B)**, *F solan*i **(C)** and mixed samples **(D)**. Control strains: *Fusarium equiseti*, *Fusarium proliferatum*, *Fusarium redolens*, *Fusarium verticillioides*, *Fusarium chlamydosporum*, *Fusarium graminearum*, *Setosphaeria turcica*, *Fusarium graminearum*, *Alternaria solani*, *Botrytis cinerea*, *Phytophthora*, *Colletotrichum gloeosporioides*.

The amplification signal was exclusively detected in positive samples of *F. oxysporum*, *F. tricinctum*, and *F. solani*, whereas no amplification products were observed in either sterilized ddH_2_O or non-target control strains (**Figure 3**), demonstrating high specificity of the established multiplex TaqMan real-time PCR assay.

### 3.4 Sensitivity of the multiplex TaqMan real-time PCR assay

**Figure 4:**
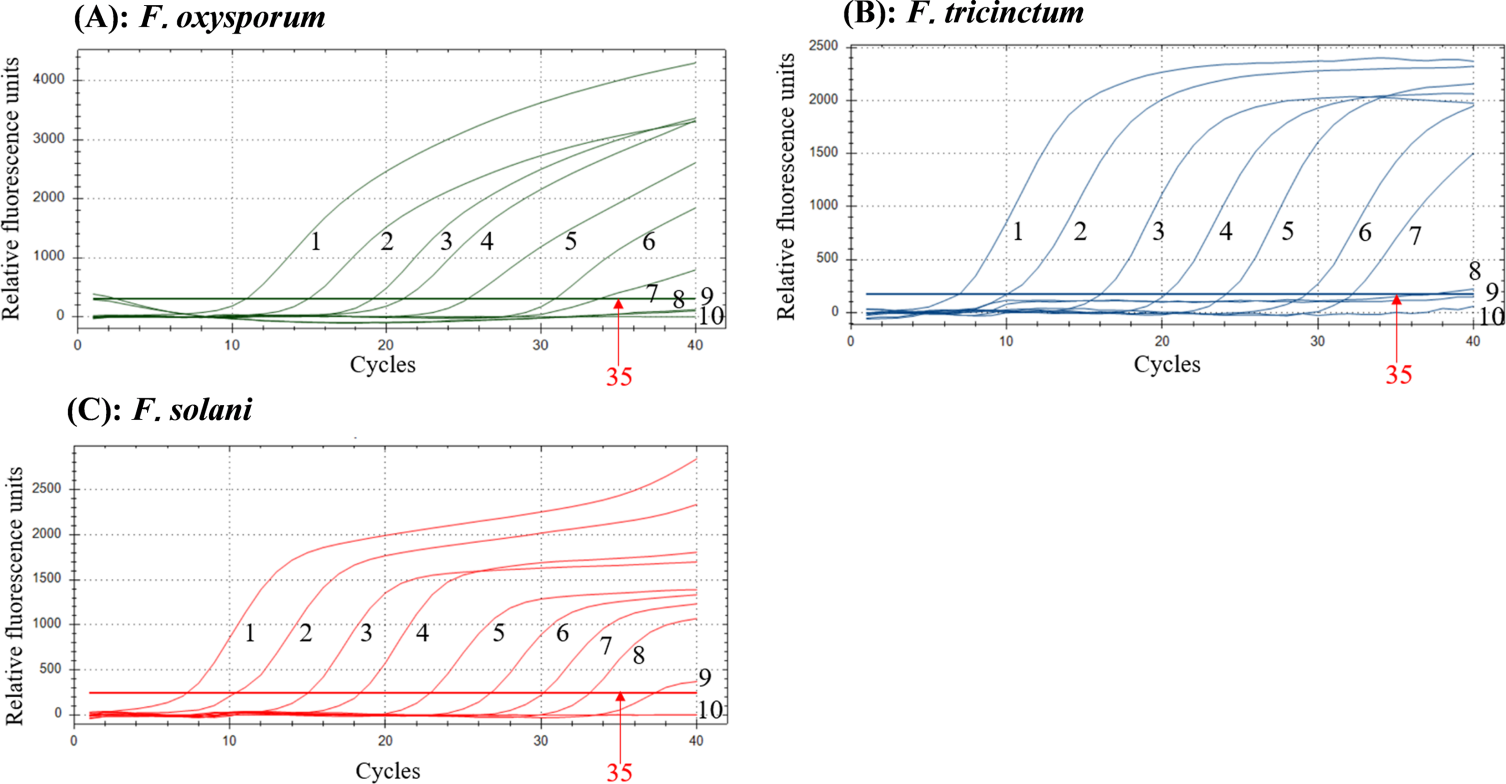
Sensitivity results of the multiplex TaqMan real-time PCR assay for *F oxysporum***(A)**, *F tricinctum***(B)**, and *F solani***(C)**. 1–10 correspond to ten-fold serial dilutions of plasmid standards for *F oxysporum*, *F tricinctum*, and *F solani*, covering concentration ranges of 1.46 × 10^9^ to 1.46 × 10^0^, 7.17 × 10^9^ to 7.17 × 10^0^, and 1.76 × 10^9^ to 1.76 × 10^0^ copies·µL^-1^, respectively.

The sensitivity analysis revealed detection limits of 1.46 × 10³ copies·μL^-1^ for *F. oxysporum*, 7.17 × 10³ copies·μL^-1^ for *F. tricinctum*, and 1.76 × 10² copies·μL^-1^ for *F. solani* (**Figure 4**).

### 3.5 Repeatability analysis

We selected standard plasmids at three concentrations (10^7^, 10^5^, and 10³ copies·µL^-1^) to evaluate both intra- and inter-assay repeatability. The resulting coefficient of variation (CV) values were predominantly below 1%, with a minority ranging between 1% and 2% (**Table 8**). These low CV values confirm the high repeatability and reliability of the established assay.

**Table 8:** Reproducibility assay of the multiplex TaqMan real-time PCR.

Standard plasmid	Concentration (copies·µL^−1^)		Intra-Assay			Inter-Assay		
			Mean	SD	CV%	Mean	SD	CV%
*F. oxysporum*	1.46×	10^7^	18.89	0.13	0.69	19.05	0.07	0.37
		10^5^	25.46	0.21	0.81	25.56	0.28	1.09
		10^3^	32.49	0.34	1.03	32.74	0.16	0.50
*F. tricinctum*	7.17×	10^7^	16.18	0.07	0.41	16.38	0.20	1.22
		10^5^	24.13	0.09	0.36	24.35	0.21	0.87
		10^3^	32.50	0.17	0.52	32.45	0.29	0.88
*F. solani*	1.76×	10^7^	14.60	0.05	0.34	15.07	0.17	1.13
		10^5^	22.88	0.11	0.48	22.97	0.07	0.31
		10^3^	29.79	0.09	0.32	30.30	0.44	1.46

### 3.6 Association between soil *Fusarium* content and root rot disease index of red kidney bean revealed by logistic regression

**Figure 5:**
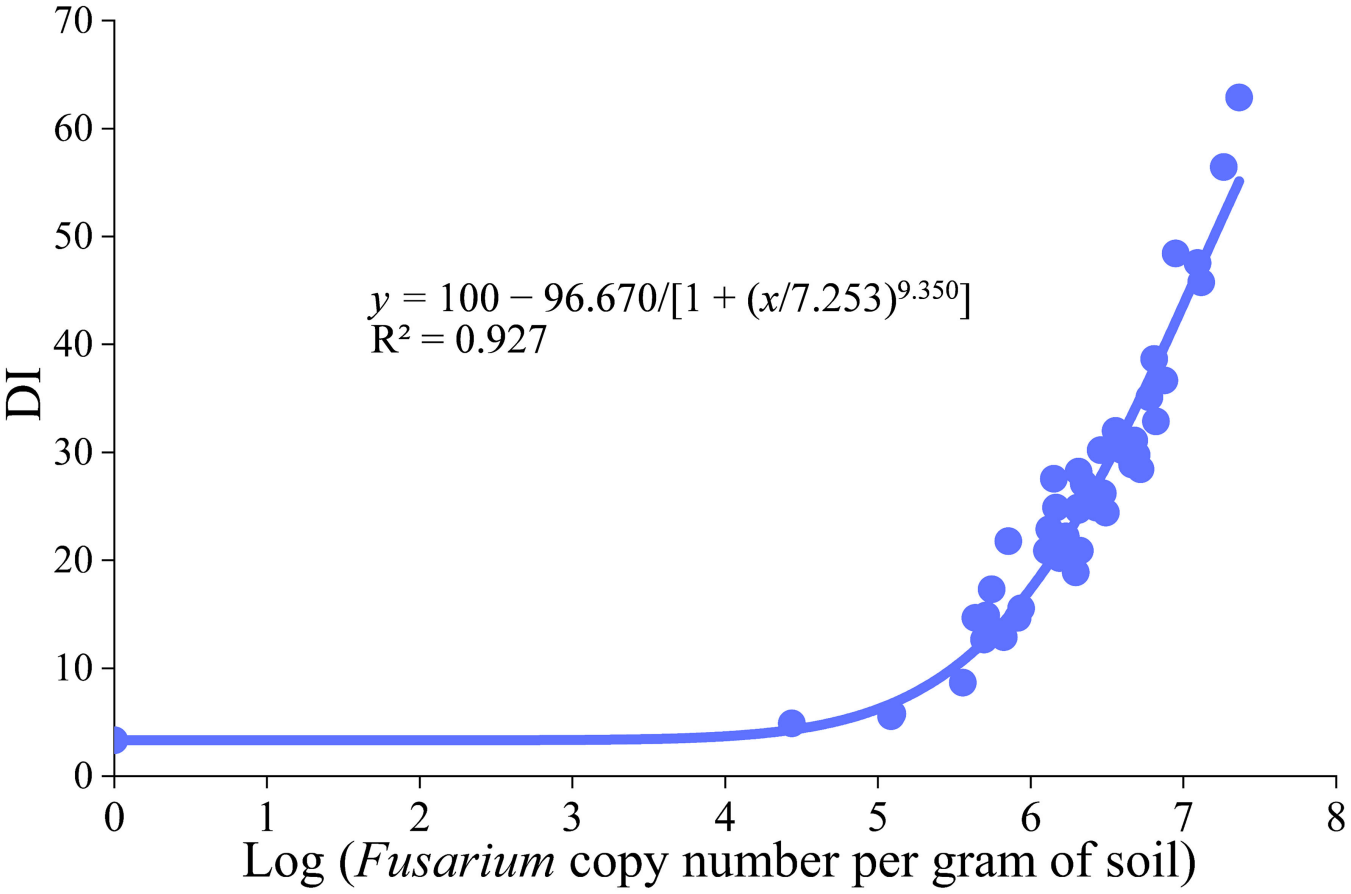
The relationship curve of disease index and *Fusarium* content in soil.

Multiplex TaqMan real-time PCR was performed on 45 rhizosphere soil samples collected from red kidney bean fields (**Table 9**). Logistic regression analysis revealed a significant correlation between the total copy number of three *Fusarium* species in soil (x-axis) and the root rot index (y-axis) (**Figure 5**), y = 100 − 96.670/[1 + (x/7.253)^9.350^], and R^2^ = 0.927. This indicates that detecting the content of *Fusarium* in soil can predict the occurrence of plant diseases to a certain extent.

**Table 9:** The results of multiplex TaqMan real-time PCR detection of field soil samples.

Samples	Cq_1_ value	Cq_2_ value	Cq_3_ value	Disease index	Samples	Cq_1_ value	Cq_2_ value	Cq_3_ value	Disease index
1	29.95	29.97	–	31.11	24	31.37	30.76	–	24.89
2	32.05	31.34	30.14	28.22	25	31.64	30.59	29.80	24.44
3	32.50	32.02	30.42	27.56	26	32.22	31.51	33.52	22.22
4	28.83	27.59	28.55	56.44	27	33.19	–	–	5.78
5	29.54	28.14	28.62	45.78	28	–	–	33.11	4.89
6	30.83	29.68	29.61	28.44	29	–	–	–	3.33
7	31.41	–	29.49	21.78	30	–	33.80	–	8.67
8	30.49	29.96	30.40	28.89	31	36.29	33.27	35.91	12.67
9	31.34	35.55	35.14	14.67	32	33.22	–	–	5.56
10	29.83	–	31.68	20.89	33	30.70	29.77	30.66	29.78
11	35.59	–	27.88	15.56	34	–	31.43	32.69	20.22
12	–	30.95	30.04	27.11	35	35.47	33.89	30.33	14.89
13	30.23	29.62	28.58	35.11	36	31.53	30.62	31.04	26.22
14	31.67	30.62	30.51	26.22	37	30.23	–	28.58	24.89
15	32.42	32.19	30.43	22.89	38	33.11	31.13	30.46	20.89
16	29.63	32.97	–	18.89	39	30.51	–	35.31	14.67
17	36.65	32.78	35.77	12.89	40	31.22	30.88	29.51	30.22
18	31.12	30.35	28.51	30.22	41	28.11	30.44	–	32.89
19	–	28.64	28.15	48.44	42	30.44	29.17	28.04	36.67
20	29.29	28.29	28.66	47.56	43	29.97	29.40	30.30	38.67
21	29.17	–	30.46	24.89	44	26.44	28.35	27.02	62.89
22	31.99	31.43	29.69	24.67	45	35.12	–	28.55	17.33
23	30.64	30.35	–	32.00					

“–” means no Cq value; “Cq_1_ Value” means Cq value of *F. oxysporum*; “Cq_2_ Value” means Cq value of *F. tricinctum*; “Cq_3_ Value” means Cq value of *F. solani*.

## 4 Discussion

Root rot, a severe soil-borne disease in red kidney bean, is primarily caused by a complex of *Fusarium* spp. The diversity of pathogenic species involved poses considerable challenges for disease control and often leads to substantial yield losses. In recent years, the prevalence of *Fusarium* root rot has increased steadily and expanded into major production zones, including Shanxi Province. Current diagnostic approaches, however, still depend on conventional techniques that are labor-intensive, inefficient, and lack the capability to concurrently detect and distinguish these three pathogens, greatly limiting rapid identification and timely implementation of control measures (Tian et al., 2020). Therefore, establishing a specific, rapid, and simultaneous molecular detection method for the three pathogenic fungi causing root rot in red kidney bean is essential.

Although various molecular techniques—including multiplex PCR, LAMP, and real-time PCR—have been employed for *Fusarium* detection (Yang et al., 2015; Xiao and Li, 2021; Wang et al., 2023b), a multiplex TaqMan real-time PCR assay specifically targeting the dominant *Fusarium* pathogens in red kidney bean had not been established. To address this gap, we developed a multiplex TaqMan real-time PCR assay for simultaneous detection of *F. oxysporum*, *F. tricinctum*, and *F. solani*, which demonstrated high sensitivity with detection limits of 1.46 × 10³, 7.17 × 10³, and 1.76 × 10² copies·μL^-1^, respectively. The observed variation in detection limits among the different fungal species may be largely attributed to inherent differences in the binding efficiency and amplification specificity of the primers and probes designed for each target, which directly contribute to the differences in detection limits. Additionally, the sensitivity of the present method is on par with that reported by Carneiro et al. (2017), who developed a TaqMan assay for detecting *Fusarium fujikuroi* in rice, with a detection limit of 3.0 × 10² copies·μL^-1^, as well as with the assay by Campos et al. (2023) targeting *Fusarium* species in tomato, which exhibited a detection threshold of 2.29 × 10² copies·μL^-1^. Our assay also exhibited excellent reproducibility, with both intra- and inter-assay coefficients of variation (CVs) below 2%. The use of plasmid-standard curves enabled absolute quantification of pathogen biomass, providing a reliable basis for correlating pathogen DNA concentration with disease severity. However, it should be noted that plasmid standards may not fully simulate the complexities of natural soil matrices, including variations in nucleic acid extraction efficiency and amplification inhibitors. Future studies could further validate and refine quantification accuracy using naturally infected soil samples with varying organic matter contents.

The high sensitivity of this assay is especially critical for early detection. Infected red kidney bean plants often exhibit no or minimal aboveground symptoms before significant root damage occurs, rendering visual diagnosis unreliable and frequently resulting in missed opportunities for timely intervention. The multiplex TaqMan assay developed in this study, combined with the established logistic model correlating soil *Fusarium* DNA concentration (load) with the disease severity index (DI), provides a powerful tool for early disease prediction. This approach enables the proactive implementation of targeted control strategies before visible symptoms appear and crop losses escalate. The integration of pathogen detection with quantitative modeling in this study supports an effective “detection-prediction” strategy for managing red kidney bean *Fusarium* root rot. This approach may provide a key tool to shift management from reactive, symptom-based control toward proactive, data-driven strategies. Specifically, its practical value is twofold: it enables early warning and defines actionable intervention thresholds. Consequently, it facilitates more precise and timely control measures, advancing the implementation of IPM strategies.

It is important to note, however, that the current logistic regression model does not account for potential variations in virulence among the three *Fusarium* species or possible synergistic or antagonistic interactions between them. These factors are of significant importance for further refining and optimizing the predictive accuracy of the red kidney bean root rot model and should be considered key directions for future research.

## 5 Conclusions

In conclusion, we developed a highly sensitive and specific multiplex TaqMan real-time PCR assay for the simultaneous detection of *F. oxysporum*, *F. tricinctum*, and *F. solani*. This robust molecular tool not only facilitates routine pathogen diagnosis and epidemiological surveillance but also, when integrated with the predictive logistic model, enables pre-symptomatic disease risk assessment. Collectively, this approach provides a foundation for implementing targeted control strategies against *Fusarium* root rot in red kidney bean, thereby contributing to reduced yield losses and more sustainable agricultural practices.

## Data Availability

The original contributions presented in the study are included in the article/supplementary material. Further inquiries can be directed to the corresponding author.
